# The story of antipsychotics: Past and present

**DOI:** 10.4103/0019-5545.58304

**Published:** 2009

**Authors:** Chaitra T. Ramachandraiah, Narayana Subramaniam, Manuel Tancer

**Affiliations:** Rajiv Gandhi University of Health Sciences, Bangalore, India; 1 M.S. Ramaiah Medical College Hospital, Bangalore, India; 2 Department of Psychiatry and Behavioral Neurosciences, Wayne State University School of Medicine, Detroit, MI, USA

Antipsychotics have been broadly classified into classical/typical, which includes phenothiazines and butyrophenones, and atypical antipsychotics, which includes benzamides. The psychiatric effects of all the progeners of these groups were discovered serendipitously. Chlorpromazine, belonging to phenothiazines, heralded the “psychopharmacological era” and replaced biological therapies such as electroconvulsive therapy, insulin coma, frontal lobotomy and simple sedation, causing an important revolution in psychiatric practice. Derivatives of phenothiazines, such as methylene blue, had been used since the nineteenth century in the dyeing industry (textile as well as histopathological preparations) and pharmaceuticals such as antiseptics and antihelmenthics.

A group of phenothiazine derivatives with an aminate chain were synthesized by a team led by Paul Charpentier to exploit the antihistaminic properties as pre-anesthetic medication. In 1949, Henri-Marie Laborit, a French army surgeon, used promethazine, a phenothiazine derivative, along with barbiturates, to prevent surgical shock and called this “lytic cocktail.” He also noticed that the patients who were extremely anxious were made calm, relaxed and indifferent to the surroundings. Agitated patients got subdued and co-operative as if they had a “pharmacological lobotomy.”

The research on phenothiazines continued until 1950, when a chlorinated derivative of promazine was developed: RP-4560, later named chlorpromazine, which not only had antihistaminic properties but was also adrenolytic, gangliolytic and antiemetic among many other effects. Laborit tried to convince psychiatrists about the therapeutic uses of the drug in psychiatry, in line of his hypothesis and observations, especially in sleep disorders. No one in the scientific community was enthused about it. Finally, Joseph Hammon tried it for the first time in 1952 on a manic patient. The patient not only calmed down but was also able to maintain this state. He was eventually discharged after 3 weeks. Researchers almost disregarded this effect because the patient was on other medication, such as barbiturates, opiates and electro-convulsive therapy. In the same year, Jean Delay and Deniker conducted a study using chlorpromazine alone and observed a group of symptoms with decreased motor activity and affective indifference. They named the constellation of symptoms as “neuroleptic syndrome.” The word neuroleptic was suggested by Jean Delay in 1955, which meant “that take the nerve.” The duo further went on to present six clinical reports on 38 patients with mania and psychosis and confirmed the effectiveness of chlorpromazine. Heinz Lehmann, a psychiatrist from Berlin and a refugee residing in Canada, published similar articles on the use of chlorpromazine exhaustively over several years. He used the drug in different psychiatric conditions with psychomotor agitation (acute and chronic mania, schizophrenia, senile psychosis, post lobotomy and mentally challenged). He obtained positive results in 66%, but the response was the best in patients with manic depression, who were manageable within 24 hours, subsequently, having fewer relapses. He also cautioned that, in patients with chronic schizophrenia who were nonresponders, the possible toxic effects of the drug prevailed. In 1954, Elkes and Elkes conducted a historic study on psychotic patients, which was randomized and placebo-controlled, showing the effectiveness of chlorpromazine. Eventually chlorpromazine started being accepted by psychiatrists all over the world, although a slow start. Thus, it paved the way for the “neuro-biological” basis of psychiatric illnesses.[1]

Haloperidol belongs to butyrophenones and was synthesized in 1958 at a Belgian laboratory by Paul Janssen. Janssen laboratories were then trying to develop a more powerful analgesic than dextromoramide. They used pethidine, the byproducts of which were named butyrophenones and haloperidol was the 45^th^ of the series, R-1625. They observed that it was poorer an analgesic compared with opioids. However, the experimental mice were sedated and went into a cataleptic state similar to that produced by chlorpromazine, after an initial state of excitation. First clinical studies were conducted by Bloch on patients with delirium tremens. It resulted in no significant sedation except for hypotension. After a few weeks of its synthesis, the drug was used intravenously on a psychiatric patient with an emotional crisis by a resident physician Pinchard. The patient became considerably calm and later went into a state of sedation. Studies conducted all over Belgium with this drug showed outstanding results in agitated patients but its hallucinolytic effects surpassed the others. Delay and Deniker conducted studies in France on this drug as well and obtained similar results. However, they warned of its extrapyramidal side-effects similar to the effects of chlorpromazine. Haloperidol has also been considered very efficient in paranoid states.[2]

After the advent of chlorpromazine, other drugs such as haloperidol, trifluperazine, thioridazine and fluphenazine came into use. All were found to have comparable efficacy, but had serious neurological side-effects such as neuroleptic malignant syndrome. The search continued for a neuroleptic with lesser side-effects. In 1958, a group of tricyclic compounds was synthesized, based on the antidepressant imipramine in a Swiss laboratory. Some of these compounds were found to have neuroleptic properties. One of them was clozapine. The initial studies by Wander in 1959 obtained mixed results, but one of them was significant; clozapine did not cause catalepsy in animal studies. In 1962, Gross and Langner conducted trials with human subjects and found the drug to be ineffective for psychosis. In 1966, Hanns Hippius, a German researcher, was asked to continue the trials on humans by Wander. The results confirmed that clozapine was an effective antipsychotic with no disabling neurological side-effects. The psychiatric community remained sceptical because the drug lacked side-effects. They believed that if there are no Parkinsonian symptoms, the drug is not a true antipsychotic. The more pronounced the extrapyramidal symptoms were, the more effective the drug was. Despite this belief, there were many patients with schizophrenia that were placed on clozapine.

Finally, after several large trials summarized by Stille and Hippius, clozapine was launched into the market in several European countries. In 1973, Gilbert Honigfeld, a clinical psychologist, started open-label phase-2 trials on prison inmates in the USA. The inmates experienced increased heart rate and syncope due to orthostatic hypotension even with lower doses (25-75 mg). This finding was never mentioned in the earlier literature. The solution was to titrate the dose slowly up to a tolerable level, starting from a smaller dose. In 1974, open-label studies were conducted on schizophrenic patients by Simpson and Varga and double-blind clinical trials by Shopsin, Klein, Aaronson and Collora. Later, many multicentered trials of clozapine vs. chlorpromazine were conducted. When clozapine was gradually being accepted as a promising antipsychotic, an adversity befell upon it. In 1975, the Journal, Lancet, reported that 18 patients had developed severe blood dyscrasia. Sixteen of them had agranulocytosis with nine deaths. This was reported from Finland by Idanpaan- Heikkila, Alhava, Olkinvoura and Palva. The government agencies then ordered clozapine to be removed from the market in Finland as well as the other European countries. Later studies all over the world showed that agranulocytosis was 20-times higher in south-western Finland compared with the rest of the world. The exact causes for this Finnish “epidemic” were not known. In the meanwhile, agranulocytosis and sudden death with chlorpromazine and other phenothiazines were also reported. Shopsin *et al*. reported that the worldwide incidence of agranulocytosis with clozapine (0.3%) was similar to chlorpromazine (0.1-1.0%). However, there were controversial findings in some of the later studies. Amsler, Teerenhovi, Barth, Harjula and Vuopio did a thorough analysis and found that agranulocytosis occurred within the first 3 months, whether fatal or not, and deaths were due to secondary infection and delays in the detection or lack of preventive measures. This fact led to the mandatory weekly checks of white cell counts in the blood for the first 18 weeks. Until then, clozapine was restricted to compassionate use for treatment-resistant cases of schizophrenia. Later on, the worldwide support for clozapine grew and was also reported to be used in children with Tourette's syndrome successfully by Caine, Polinsky, Kartzinel and Ebert. In 1984, a 6-week double-blind study was conducted at 16 sites in the USA comparing clozapine with chlorpromazine. Clozapine was shown to have a definite superiority over chlorpromazine. The improvement not only continued even after 6 weeks, but the drug worked for positive as well as negative symptoms in schizophrenia. Kane, Henigfeld, Singer and Meltzer, finally, were able to show clozapine to be an effective antipsychotic without extrapyramidal symptoms and hence named it “atypical.” Although it had other benign side-effects, such as drowsiness, hypersalivation, hypotension and a propensity to decrease seizure threshold, clozapine continues to be the drug of choice for treatment refractory schizophrenia.[3]

Although the process of scientific drug development is slow and tedious, clozapine still emerged victorious. The 1990s was the decade that witnessed the advent of several other atypical antipsychotics, such as risperidone, olanzapine, quetiapine, ziprasidone and similar drugs. These were considered as effective as the other antipsychotics and had the least propensity to cause extrapyramidal side-effects, but had other side-effects innate to the group, such as metabolic syndrome. While we compare the typical antipsychotics with the atypicals in terms of their effectiveness and side-effect profile, none seems to be more superior compared with the other. We have come a long way from insulin therapy and prefrontal lobotomies to compounds that not only are effective antipsychotics but also help patients to lead a better life, despite some of the side-effects. The search continues to develop drugs that address all aspects of the illness, the positive, the negative and the cognitive symptoms, and for that “ideal” antipsychotic.
